# Casting of carbon cloth enrobed polypyrrole electrode for high electrochemical performances

**DOI:** 10.1016/j.heliyon.2019.e03122

**Published:** 2020-01-21

**Authors:** Rama Devi, Kavita Tapadia, Tungabidya Maharana

**Affiliations:** Department of Chemistry, National Institute of Technology, Raipur, Chhattisgarh, India

**Keywords:** Electrochemistry, Nanotechnology, Polypyrrole, Electrochemical studies, Scanning electron microscopy, Supercapacitors

## Abstract

The present investigation deals with the fabrication of a novel flexible and highly conductive PPy electrode. This was made by festooning PPy nanoparticles on carbon cloth (CC) by using chemical liquid process. The developed porous PPy@CC composite have good flexibility with low weight (1.1 mg) and high electrical conductivity (89 Ω^−1^cm^−1^). Fourier-transform infrared spectroscopy (FTIR) and X-ray diffraction spectroscopy (XRD) confirmed the formation of PPy on carbon cloth. Scanning electron micrographs (SEM) reveals that the PPy nanoparticles are encapsulated in carbon cloth. The fabricated carbon cloth has been used for solid-state symmetrical supercapacitors (SC) and low-cost material for electrode in potential energy storage devices. These film electrodes showed much superior electrochemical performance i.e. high stability under different current density, encouraging energy density, lower internal resistivity and higher specific capacitance. Synthesized flexible PPy@CC composite electrodes have brilliant applications in various kinds of electrochemical energy storage devices.

## 1 Introduction

The design and development of inexpensive, thin, flexible, light weight and even roll up portable electronic devices with multifunctional applications have recently received overwhelming interest among researchers from academia and industry. This warrants innovative technology for developing energy storage devices having less mass and has flexibility endowed with high energy and power density even under bending conditions [1]. The two important electrical energy storage systems are Batteries and Supercapacitors. Supercapacitors are superior to batteries in terms of their enhanced specific capacitance [2], better cyclic stability [3], high power and energy density [4], environmental benignancy and safety [5].

Various technologies such as micro fabrication technology open up low-cost production for a high performing, robust and adjustable micro supercapacitor system which further explores its feasibility to be integrated into miniaturized devices [6,7]. Although the theoretical performance of these flexible energy storage devices is very high, their efficiency is extremely retarded owing to the assembly of various components in the device. This will greatly affect the commercialization of these portable electronic products [8]. Hence intense care has to be taken for the preparation of efficient, flexible, highly capable superior performance energy storage devices. Generally some components of supercapacitors are as electrodes, electrolytes, separators and current collectors. Enormous efforts have been made for the past two decades, towards the development of nanostructure electrode materials. The study of materials for electrode with good electrical conductivity and stable performance has been a great research topic. Construction of carbon cloth based supercapacitors with a reasonably better performance at a low cost is highly beneficial, since carbon cloth is composed of large number of fibers having high surface area with unique porous bulk structure along with rough and absorptive surface properties [9].

In general, carbon based materials such as carbon nanotubes, graphene, activated carbon etc. along with transition metal, transition metal oxide such as TiO_2_, RuO_2_, NiO, MnO_2_, Co_3_O_4_, V_2_O_5_ have been used commonly as electrode resources for supercapacitor due to their low cost, good chemical stability, electronic, optical and UV absorbing properties. They have been used for a variety of applications but its usage in the field of electronics is limited by its low σ [10]. The wide band gap of metal oxide results in an excellent transparency to visible light but conductive polymers have many potential applications due to their combined electrical conductivity and polymeric properties such as flexibility, low density and facile structural modifications [11,12]. One of the interesting features of these intrinsically conducting polymers is that it is possible to tune the electrical conductivity (σ) over a wide range from insulating to metallic by doping, addition of fillers and intercalating polymers into different inorganic host materials [13].

Conducting polymers such as polypyrrole, polyaniline, polythiophene, polyethylene, polyphenylvinylene etc. have fascinated extensive investigation in the field of electrode materials of supercapacitors. Among the various conducting polymers, polypyrrole is much exciting material for supercapacitors due to its superior properties such as easy synthesis [14], high electrical conductivity [15], cheaper, high mechanical stability [16] and good environmental firmness [17]. Porous PPy is one of the most essential materials, especially for applications such as energy storage [18], sensor [19], organic light-emitting diode (OLED) [20], solar cell [21], flexible battery [22], catalysts [23] & radio frequency identification (RFID) tags [24] etc. Recently, PPy nanoparticles (PPyNPs) have been used in many electrochemical devices as conductive dopant in electrode materials.

Carbon cloths are attractive alternative for application as flexible electrodes due to their good electrical conductivity, light weight, chemical stability, high porosity and flexibility. In addition, CC also displays adequate electrochemical properties [25].

On the current investigation, our approach requires no binders or conductive agents, so it is possible to avoid common problems resulting from their residues that lead to reduced electron storage capacity and inconsistent cycle life in conventional PPy power based supercapacitors. A simple and cost-effective technique is being used in the present work to deposit PPy nanostructures on the carbon cloth surface. The resulting hierarchical electrode with strong adhesion between hybrid spherical shaped PPy and flexible CC not only delivers promising capacitive performance due to rapid penetration of electrolytes, but also allows the electrode to bend without decreasing capacitance by releasing the bending stress by adjusting the interparticle separation.

Thus, the present investigation aimed at development of a general approach for fabricating PPy nanoparticles exhibiting excellent performance. On carbon cloth, the PPy nanoparticles grow directly as "core" by themselves without any binder as additives that ensure their high electrical conductivity.

## 2 Experimental section

### 2.1 Materials

Pyrrole monomer and sodium dodecyl sulphate (SDS) were purchased from Sigma Aldrich. Pyrrole was distilled to separate out the polymerized components and a colorless liquid obtained was stored at 0 °C for further use. Ferric chloride (FeCl_3_) purchased from Merck limited, Mumbai and Nitric acid purchased from Loba Chemie, Mumbai, India were used as received. All these solvents were analytical grade. Carbon cloth (thickness - 0.2 mm) was purchased from Hitsan Incorporation, Chandigarh India.

### 2.2 Synthesis of the PPyNPs electrode

Carbon Cloth (CC) enrobed Polypyrrole Electrode was made up by simple in situ chemical polymerization [26]. The square substrates (1 × 1 cm^2^) of CC were refluxed in 6 M nitric acid at 80 °C for 6 h. Treated substrates were dipped in a mixture solution of 0.1ml of distilled pyrrole and 0.416g of SDS dissolved in 20 ml anhydrous alcohol and was kept in ice bath for 3 h. Then the CC was removed and dried for 1 h. Further, the CC was dipped in 20 ml (0.036M) FeCl_3_ solution and was stirred for 1h. Finally, the CC enrobed PPy was washed with ethanol followed by deionized water and was dried at 40 °C for 12 h to remove the unreacted monomer. According to this paper [27], the prepared PPyNps in this concentration are good electrochemical features such as high specific capacity, good cyclic stability, high charge-discharge stability, etc., but we have changed oxidant (FeCl_3_) and surfactant (SDS) to improve their conductivity and electrochemical properties.

### 2.3 Preparation of electrolytes

PVA- H_2_SO_4_ electrolytes were prepared as per the reported method [28] for the invention of energy storage devices. A mixture of 6g PVA powder and 6g of H_2_SO_4_ were added in 60 ml deionized H_2_O and was stirred at 85 °C to obtain a clear solution and cooled to room temperature for further use.

### 2.4 Fabrication of solid state symmetrical supercapacitors

A piece of Whatman filter paper dipped in PVA- H_2_SO_4_ electrolyte for 30 min was used as separators for supercapacitors. This was sandwiched between the two CC enrobed PPy electrodes and was kept in a fume hood for several hours at room temperature to remove the excess electrolyte.

## 3 Materials characterization

The morphology and structure of the CC enrobed PPy electrodes were acquired using Scanning Electron Microscope (ZEISS EVO-50 SEM machine). EDX (Energy Dispersive X-ray) was also observed. Fourier Transform Infrared (FTIR) spectra of prepared samples were recorded by an Agilent Technologies Cary 630 FTIR spectrophotometer. XRD data of CC enrobed PPy electrodes were obtained from X-ray diffractometer of PANalytical XPERT–PRO with Cu-Kα radiations at scanning range of 0°–80°. Raman spectrums of prepared nanoparticles were observed by Research India RIAR-0403 spectrometer RIRAMAN532. The conductivity of the CC enrobed PPy electrodes were measured by a standard four-probe conductivity meter using a SES Instrument make CCS-01 programmable current source and a DMV-001microvotameter. The electrical conductivity (σ) was calculated as follow:

(1) $$Po=\frac{V}{I}2\pi s$$

(2) $$\sigma =\frac{1}{Po}$$

where Po represents resistivity, V represents voltage, I represent current and s represents probe distance.

### 3.1 Electrochemical measurements

The electrochemical properties of the as-prepared CC enrobed PPy electrodes were carried out on a Metrohm Autolab (MULTI AUTOLAB M204). Prepared electrodes were used as the working electrode, Saturated Calomel Electrode as reference electrode and platinum metal was used as counter electrode. The specific capacitance (C_s_) was calculated by as follow:

(3) $$Cs=\frac{I\times \Delta t}{m\times \Delta v}$$

where Δ*t* represents discharge time, Δ*V is* discharge voltage, I represent current and *m* is mass of electrode material. The cyclic voltammetry (CV) and galvanostatic charging/discharging (GCD) techniques were engaged to study the electrochemical properties of the PPy@CC nanoparticles. The applied potential range is from -0.2 to 0.8V in a PVA-H_3_PO_4_ electrolyte. (EIS)Electrochemical impedance spectroscopy was employed with amplitude of 10mV in the frequency range between 0.01 to 100 kHz.

The energy density (E) in Whkg^−1^ and power density (P) in Wkg^−1^were calculated from the formula (4formula (4) and (5)(5)

(4) $$E=\frac{1}{2}\times Cs\times \Delta v^{2}\times \frac{1}{3.6}$$

(5) $$P=\frac{E}{\Delta t}\times 3600$$

where Cs represents specific capacitances, Δv refers to the potential window and Δt is the discharge time.

## 4 Results and discussion

### 4.1 Polymerization mechanism of PPy@CC composites

**Figure 1:**
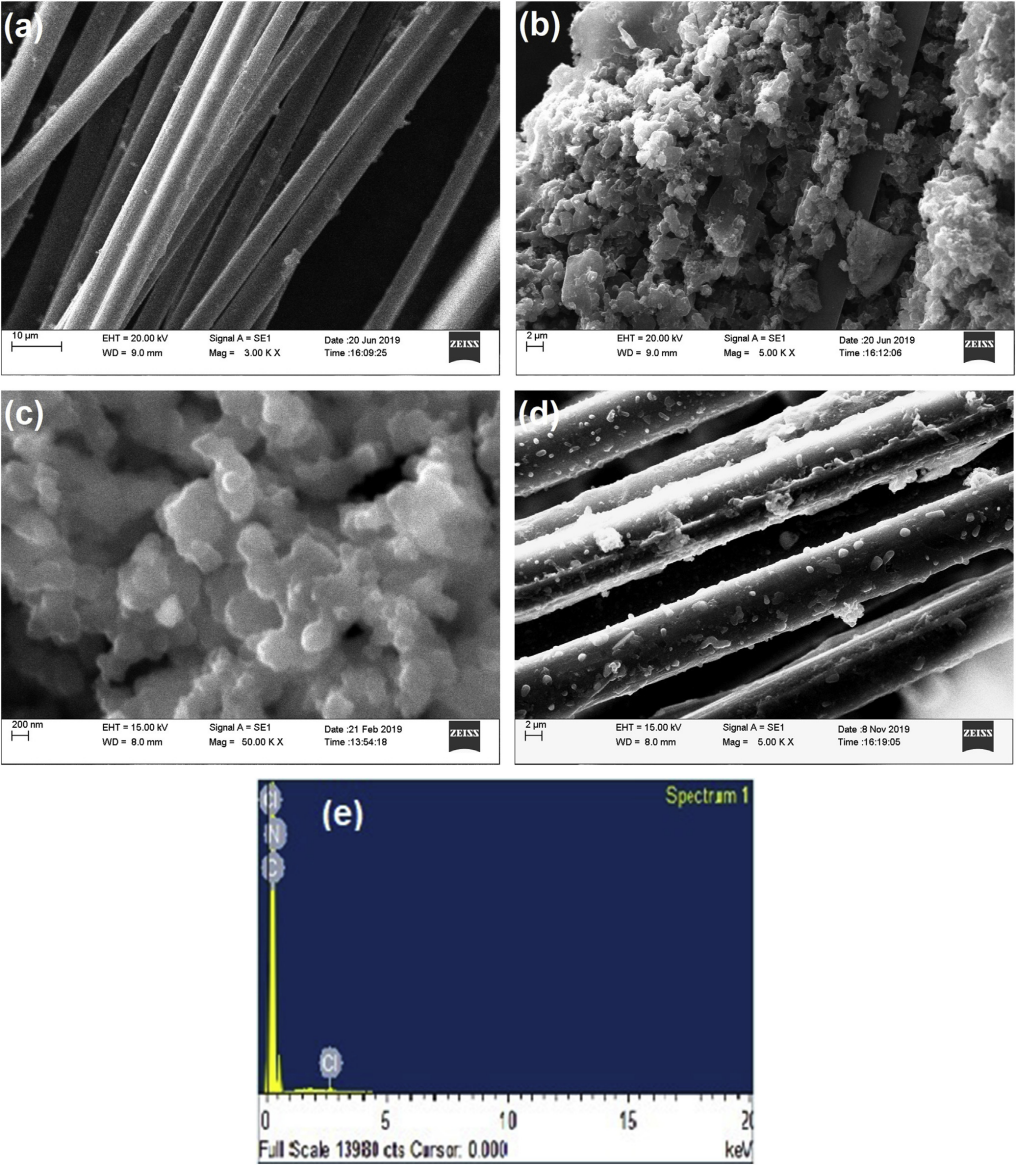
Scanning Electron Micrographs (SEM) of (a) bare carbon cloth, (b) and (c) CC enrobed PPy electrodes with different magnification before cycling test (d) CC enrobed PPy electrodes after cycling test (e) energy dispersive spectra (EDX) of CC enrobed PPy electrodes.

PPy@CC composites are manufactured using one-step chemical polymerization of pyrrole on carbon cloth substrates, using sodium dodecyl sulfate (SDS) as the “soft” template. Figure 1a-c displays the SEM images of the as-prepared PPy electrode. It can be seen that well-ordered PPyNPs were aligned vertically on the carbon fiber surfaces in the carbon cloth forming a spherical structure consisting of the three-dimensional conductive carbon skeleton and the electroactive polymer nanoparticles. Firstly, pyrrole monomer and SDS anions are absorbed on carbon fibers surfaces in the carbon cloth due to the π-π interactions between pyrrole aromatic rings as well as of SDS with the sp2 carbon [29]. These π-π interactions then provided sufficient nucleation sites for the growth of PPy NPs. FeCl_3_ acted as the oxidant that produced the chemically active cation radicals of pyrrole. The combination of two radicals led to a bond between their α positions and the creation of dihydromer dication. The aromatic dimer formed by the loss of two protons. The polymer chains continued to grow as long as pyrrole and FeCl_3_ were available [30]. The PPy chain had an ultra-long conjugated structure with alternately arranged C = C bonds and C-C bonds. In the initially formed PPy oligomers, the SDS anions doped and protect them from random polymer growth [31]. The hierarchical structure of the carbon fibers in carbon fabric are distributed in three dimensions and the upward-growing PPy nanoparticles provide an easy ion diffusion path as well as a quick electron transport avenue that is critical to high charge storage performance.

### 4.2 Characterizations of CC enrobed PPy electrodes before and after the cycling test

In situ polymerization method has been employed for preparation of PPy nanoparticles. The PPy nanoparticles get attached to the surfaces of conductive carbon cloth substrate which was confirmed by FTIR, XRD, Raman spectroscopy and SEM-EDX analysis.

#### 4.2.1 SEM and EDX analysis of polypyrrole nanoparticles

The scanning electron micrographs (SEM) of bare carbon cloth and CC enrobed PPy electrodes are presented in Figure 1a & 1b. The SEM micrographs of PPy exhibited its spherical nanometer-size nature. The PPy nanoparticles are observed to have a distribution of dimensions between 50–150 nm which shows highly interconnected network of PPy with carbon fiber and uniform coating of PPy on the surface of carbon cloth. The SEM results confirm that in situ polymerization system was successful in the synthesis of CC enrobed PPy electrodes. After the long cycling study, scanning electron microscopy (SEM) observations were carried out. It is observed that after charging-discharging cycles the aggregated PPyNPs collapse completely (Figure 1b and d). These nanoparticles have higher surface energy from the surface energy point of view than nanofilms, which tend to be more unstable [32]. The composition of CC enrobed PPy electrodes were analyzed by energy dispersive spectra (EDX). Identification lines for the large emission energies of carbon, nitrogen and chlorine are displayed in the spectrum. Here chlorine indicates the formation of polaron in polypyrrole ring which is responsible for increasing conductivity of the sample.

#### 4.2.2 FTIR spectrum of polypyrrole

**Figure 2:**
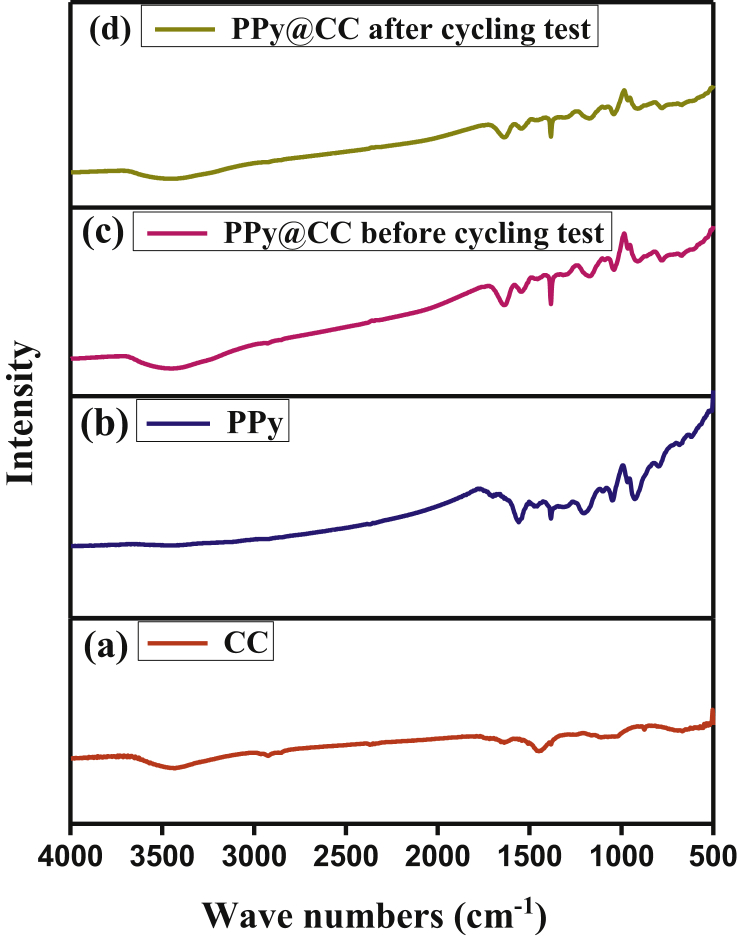
FTIR spectrum of (a) bare carbon cloth, (b) PPyNPs, (c) and (d) CC enrobed PPy electrodes before and after cycling test.

**Figure 3:**
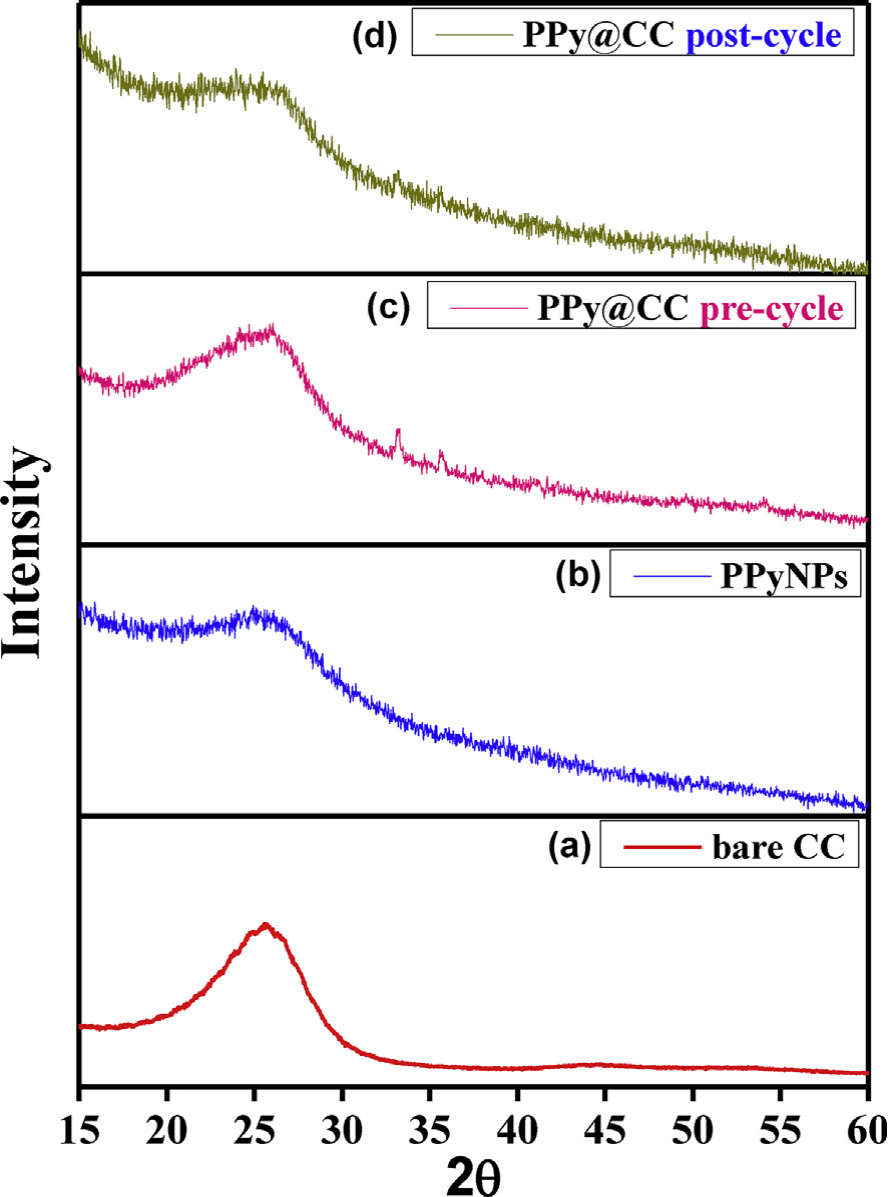
X-ray diffractograms of (a) bare carbon cloth, (b) PPyNPs, (c) and (d) CC enrobed PPy electrodes before and after cycling test.

The chemical composition of CC, PPy and CC enrobed PPy electrodes were characterized by FTIR spectroscopy. The FTIR spectrum of CC (Figure 2a) showed a large peak near 3445cm^−1^ that can be attributed to OH stretching vibration, suggesting that oxygen also exists on the surface of CC [33] and the stretching of C=C occurs at 1478 cm^−1^. The FTIR spectrum of PPy electrodes (Figure 2b) showed a strong peak near 1590 cm^−1^, 1540 cm^−1^, 1480 cm^−1^ due to pyrrole ring stretching [34] and the intense band present at 1384 cm^−1^ indicates the presence of polaron to increase the conductivity of PPy chain [34,35]. The peak at 779 cm^−1^ appears due to C-H wagging vibrations [36,37]. A broad band located at 3445 cm^−1^ is due to N-H stretching vibration [38]. A strong peak at 1300 cm^−1^& 1041 cm^−1^ for C-H in-plane stretching vibrations. The band at 1174 cm^−1^ and 911 cm^−1^ is due to the doping state of polypyrrole [39]. These all peaks are also appearing in CC enrobed PPy. Thus, FTIR spectrum confirmed the fabrication of CC enrobed PPy electrodes. After the cycling tests, very similar featured bands in FTIR (Figure 2d) are present but with decreased intensity. The peak of C-C in-ring and C-C inter-ring extending at 1590 cm^−1^ remains exceptional in the spectrum of CC enrobed PPy. This means that the repeated charging-discharging (consistent with Figure 3b) does not break the CC enrobed PPy backbone structure and therefore ions can travel smoothly. Since the conductivity of conductive polymers is determined primarily by the duration of conjugation and the degree of doping [32] those disappeared in-ring bands of CC enrobed PPy after cycles that indicate a decreases in conductivity.

#### 4.2.3 X-ray diffraction analysis

A typical X-ray diffraction pattern of CC, PPy and CC enrobed PPy electrodes are shown in Figure 3. The all XRD patterns display large peaks in the 15° < 2ϴ < 30° area, which is attributable to the interplanar d spacing of 3.45 Å [40] showing that the resulting polypyrrole powders are amorphous in nature [41,42], which is due to the π-π interaction of the PPy chain [26,43,44]. After cyclic test, the CC enrobed PPy shows a similar diffraction peak at 26°. These imply a more ordered PPy enrobed structure by the CC. This shows that molecular chains of CC-coated PPy are aligned and oriented much more regularly [32].

#### 4.2.4 Raman spectrum of polypyrrole

**Figure 4:**
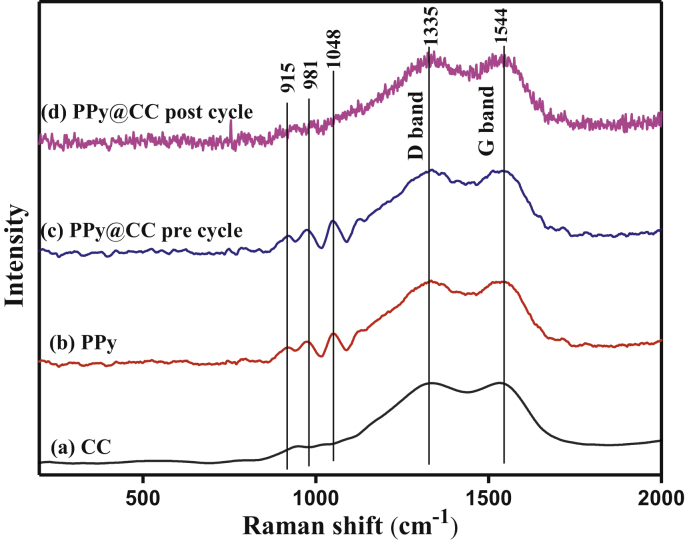
Raman spectrum of (a) bare carbon cloth, (b) PPyNPs, (c) and (d) CC enrobed PPy electrodes before and after cycling test.

The compositions of the CCCCC enrobed PPy products were characterized by Raman spectroscopy and the presence of PPy can be clearly reflected in the Raman spectrum. As shown in Figure 4, it is possible to observe the representative Raman bands from the carbon cloth at 1335 (D band) and 1544 cm^−1^ (G band). After PPy coating, the Raman peaks appear at 915, 981, and 1045 cm^−1^ [45].

#### 4.2.5 Conductivity measurement

The conductivity of the pure CC is 6.23 S/cm and that of enrobed PPy is as high as 89 S/cm. The higher conductivity of CC enrobed PPy electrodes is because of the formation of nano sized and ordered PPy particles due to the presence of sodium dodecyl sulfate (SDS) which acts as an anionic surfactant. It is to be noted that the conductivity of CC enrobed PPy electrodes is much higher in the present study. After cyclic test their conductivity is decreses up to 76.32 S/cm because the concentration of PPy film on CC substrate decreases.

#### 4.2.6 Electrochemical characteristics of the CC enrobed PPy electrodes

**Figure 5:**
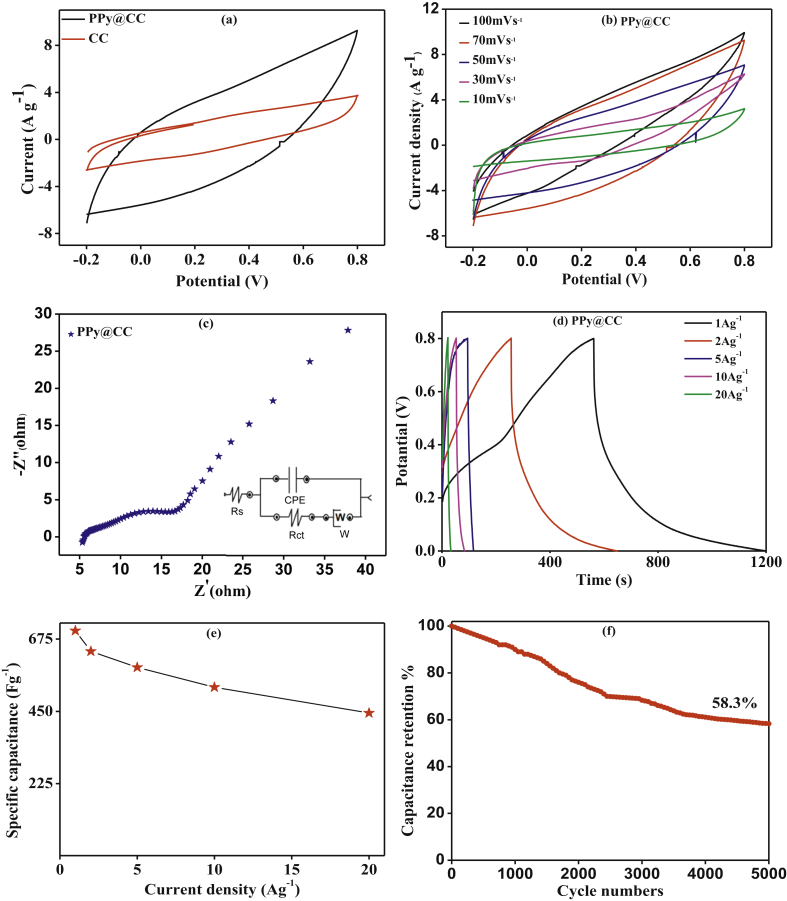
(a) CV curve of carbon cloth and CC enrobed PPy electrode supercapacitors device (SC) at 100mVS^-1^ scanning rate; (b) CV curve of CC enrobed PPy electrode SC device at different scanning rate; (c) Nyquist plots with circuit of CC enrobed PPy electrode SC device; (d) GCD curve of CC enrobed PPy electrode SC device at different current density varying from 1to 20Ag^-1^; (e) specific capacitance of CC enrobed PPy electrode SC device at various current density; (f) capacitance retention percent of CC enrobed PPy electrode SC device during 5000 cycles.

The electrochemical property of the CC enrobed PPy electrode was evaluated in a three-electrode configuration cell through cyclic voltammetry (CV), electrochemical impedance spectroscopy (EIS) and galvanostatic charge–discharge (GCD) in PVA-H_2_SO_4_ gel electrolyte. The electroactive surface area of the fabricated electrode was measured using CV by studying the effect of scan rate as a function of variation in anodic peak current. Figure 5(a) and (b) shows the CV curves of the PPy@CC electrodes at different scan rates. The CV curves reveal good symmetric and rectangular shapes, indicating enhanced supercapacitor properties, ideal capacitive performance and fast reversible Faradic reactions [46,47,48].

The synergistic effect of CC enrobed PPy electrodes is further inveterate by Electrochemical Impedance spectroscopy (EIS) measurements. Figure 5(c) shows the resulting EIS spectra for the CC enrobed PPy electrodes. All spectra are poised of a sweep in the high frequency range and a spine in the low frequency range. At high frequency, the sweep interception on the real axis reflects the corresponding equivalent series resistance (Rs) associated with the electrolyte resistance, intrinsic resistance of the active materials and the interfacial contact resistance between the active material, electrolyte and current collector [46,49]. The width of the sweep corresponds to the charge-transfer resistance (Rct) of the electrode. It can be measured that the CC enrobed PPy electrode has a smaller Rs 5.13Ω and Rct 4.76, which suggests the current formation of a CC enrobed PPy electrode and the coating has a pronounced synergistic effect between PPy and Carbon cloth.

Figure 5(d) displays the GCD curves of the flexible CC enrobed PPy electrode. All charge -discharge curves display voltage platforms as well as faradic performance verification [27]. The calculated specific capacitance is exposed in Figure 5(e). The CC enrobed PPy electrode shows improved specific capacitance of 701.3, 637.3, 587, 525.8 and 500 Fg^-1^ at 1, 2, 5, 10 and 20 Ag^−1^current densities. Furthermore, the CC enrobed PPy electrode exhibits a considerably enhanced rate capability, retaining 58.3% of capacitance after 5000 cycles at 20 Ag^-1^ (Figure 5f). Energy density is a crucial parameter which defines the capacity of electrochemical devices to store energy that serves as a power source for a longer timer. Power density is the rate of transfer of energy per mass or area and determines how quickly the energy could be discharged or charged [50]. Use Eqs. (4) and (5), the CC enrobed PPy electrode's energy density and power density are derived from the charging discharge curves. The CC enrobed PPy electrode exhibits improved energy density of 62.32 Whkg^−1^ and an encouraging power density of 399.91 Wkg^-1^.

Comparison of electrochemical performance of various electrodes has been given in Table 1. It can be seen from the data that the present work is comparable to some metal/PPy composites.

**Table 1:** Comparison of specific capacitance and cyclic stability of PPy-based electrodes.

Material	Electrolyte	Potential window (V)	Capacity (F g-1)	Current density	Capacity retention (%)	Ref.
PPy paper electrode	1 M HCL	0.0–0.8	370	1 mAcm^−1^	75.6% after cycling for 10000 times	[25]
PPy/Ni	1 M H_2_SO_4_	-0.2–0.8	488	250 mAg^-1^	56% after 1000 cycles	[37]
AgNWs/PPy	1 M KOH	0.0–0.5	509	0.31 Ag^-1^	90% after 10000 cycles	[11]
MnO_2_/PPy	0.1M Ca(NO_3_)2$4HO	0.0–1.0	290	0.5 Ag^-1^	90% after 3000 cycles	[53]
PPyNF/NiOx	6 M KOH	0.0–0.5	657	0.5 Ag^-1^	80% after 1000 cycles	[54]
PPy/MoO_3_	1M H_2_SO_4_	-0.2–0.8	687	1 Ag^-1^	83% after 3000 cycles	[52]
PPy/PbOx	PVA- H_2_SO_4_	0.0–1.2	377	2.0 Ag^−1^	94% after 5000 cycles	[55]
PPyNT/CoOx	1.0 M H_2_SO_4_	-0.2–0.8	270	1 Ag^-1^	100% after 1000 cycles	[56]
PPy	1 M KOH	0.0–0.6	773.6	1 Ag^-1^	77.6%after 1000 cycles	[51]
e-PPy	PVA/H_3_PO_4_	0.0–0.6	170	3 Ag^-1^	80.4% after 130000	[31]
CC@PPy	PVA- H_2_SO_4_	-0.2–0.8	705	1 Ag^-1^	58.3% after 5000 cycles	This work

## 5 Conclusions

A sustainable facile strategy for the manufacture of low cost, highly flexible and high-performance supercapacitors based on PPy nanoparticle decorated carbon cloth electrode has been successfully developed. The PPy nanoparticles of 150–200 nm decorated on carbon cloth electrode measured an electrical conductivity of 89 Scm^−1^. Further, fabricated supercapacitor electrode showed specific capacitance of 705 Fg^-1^, energy density of 62.32 Whkg^−1^, power density of 399.91 Wkg^−1^and outstanding cycling stability, 58.3% of its capacitance retention even after 5000 cycles at 20 Ag^-1^ current density. All these excellent properties of this sustainable flexible supercapacitor open up new window for the manufacture of flexible and wearable energy storage devices for integrating with flexible electronic gadgets. Further work is going on to include various metals in the composite and to see their effects.

## Declarations

### Author contribution statement

Rama Devi: Performed the experiments; Wrote the paper.

Kavita Tapadia, Tungabidya Maharana: Conceived and designed the experiments; Analyzed and interpreted the data.

### Funding statement

This work was supported by a fellowship provided by MHRD India.

### Competing interest statement

The authors declare no conflict of interest.

### Additional information

No additional information is available for this paper.
